# 

**Published:** 1908-09-12

**Authors:** 


### BOOKS RECEIVED.

J. and A. Churchill.
"Your Child's Health." By J. Grimshaw, M.D.

Hy. Frowde and Hodder and Stoughton.
"A System of Medicine." Vol. IV. Edited by Wm. Osler, M.D., and Thos. McCrae, M.D.

W. Green and Sons, Edinburgh.
"Recidivism: Habitual Criminality and Habitual Petty Delinquency." By J. F. Sutherland, M.D.

Methuen and Co.
"The Prevention of Tuberculosis." By Arthur Newsholme, M.D.

Reeman, Ltd.
"Death and Its Verification." By J. Brindley James, L.R.C.P.
"Infectious and Parasitic Diseases." By Millard Langfeld, M.B.

RESIDENT STAFF APPOINTMENTS.

The following gentlemen have been selected as House Officers at St. Thomas's Hospital from Tuesday, September 1, 1908 (subject to the approval of the Treasurer) :—*Casualty Officers and Resident Anæsthetists.*—A. J. S. Pinchin, B. T. Parsons-Smith, A. I. Cooke, R. W. Rix, H. T. Rossiter, and E. L. Fyffe. *Casualty Assistant.*—J. B. Mennell. *Resident House Physicians.*—C. F. O. Sankey, W. A. M. Jack, H. A. F. Wilson, J. F. Windsor, and N. W. Jenkin. *Resident House Surgeons.*—N. M. Fergusson, G. R. Girdlestone, J. L. Graham-Jones, R. G. Bingham and G. M. Huggins. *Obstetric House Physicians* (Senior) M. H. E. R. Montesole; (Junior) F. M. Neild. *Ophthalmic House Surgeon.*—J. N. Wheeler. *Clinical Assistants.*—(Throat) C. T. V. Benson, C. D. H. Corbett; (Skin) O. W. McSheehy; (Children's Surgical) C. T. V. Benson; and (Children's Medical) C. D. H. Corbett, G. H. Pridham.

### The International Congress on Tuberculosis.

The preliminary programme of this Congress, to be held in Washington from Sept. 21 to Oct. 12, 1908, has now been issued. The section meetings will take place during the week beginning Sept. 28, but the exhibition will continue open for the entire three weeks, from Sept. 21 to Oct. 12. The programme for the week includes two plenary sessions, one on Monday, Sept. 28, at which it is hoped that President Roosevelt will preside; and the other (provisionally) on Saturday, Oct. 3. Each of the seven sections into which the Congress is divided will hold two sessions daily, except on the days on which the plenary sessions take place.

In connection with the Congress a series of special lectures will be delivered in Washington and elsewhere by eminent foreigners The following are the names of the speakers and the cities in which they will lecture : Bernard Bang, of Copenhagen, Washington, Oct. 3, "Studies in Tuberculosis in Domestic Animals and What We May Learn Regarding Human Tuberculosis." A. Calmette, of Lille, France, Philadelphia, Sept. 26, "Les nouveaux procédés de diagnostic précoce de la Tuberculosis." Emil Coni, of Buenos Ayres, Washington, Oct. 2, "La Lucha contra Tuberculosis en la Republic Argentina." Arthur Newsholme, of Brighton, Washington, Sept. 29, "The Causes Which Have Led to the Past Decline in the Death Rate from Tuberculosis and the Light Thrown by this History on Preventive Action for the Future." Gotthold Pannwitz, of Berlin, Philadelphia, Sept. 24, "Social Life and Tuberculosis." R. W. Philip, Edinburgh, Boston, Oct. 6, "The Anti-Tuberculosis Programme—Co-ordination of Preventive Measures." C. H. Spronck, of Utrecht, Boston, Oct. 7. Andres Martinez Vargas, of Barcelona, New York, Oct. 9, "Tuberculosis of the Heart, Blood, and Lymph Vessels." Theodore Williams, of London, Philadelphia, Sept. 25, "The Evolution of the Treatment of Pulmonary Tuberculosis." Maurice Letulle and M. Augustin Rey (joint lecture), Washington, Sept. 30, "La Lutte contre la Tuberculose dans les grandes Villes par l'Habitation; methodes scientifiques modernes pour sa construction." L. Landouzy, of Paris, Baltimore, Oct. 5. A. A. Wladimiroff, of St. Petersburg, Washington, Sept. 28, "Biology of the Bacillus." N. Ph. Tendeloo, of Leiden, "Collateral Tuberculosis Inflammation."

We have lately received from Messrs. Allen and Hanburys, Limited, 37 Lombard Street, London, E.C., a copy of their new and greatly enlarged general price-list. This price-list is well arranged and comprehensive, and under Section VII. contains much general information that will be found useful to medical practitioners. The articles on bacterial vaccines and their preparation and on the determination of the opsonic index are special features; while the posological table of non-official drugs will be received with favour by many members of the profession. There is also a short table of the British and foreign spas, which summarises the main characters of their natural waters.

